# MRP4 sustains Wnt/β-catenin signaling for pregnancy, endometriosis and endometrial cancer

**DOI:** 10.7150/thno.32097

**Published:** 2019-07-09

**Authors:** Jun-Jiang Chen, Zhi-Jie Xiao, Xiaojing Meng, Yan Wang, Mei Kuen Yu, Wen Qing Huang, Xiao Sun, Hao Chen, Yong-Gang Duan, Xiaohua Jiang, Maria Pik Wong, Hsiao Chang Chan, Fei Zou, Ye Chun Ruan

**Affiliations:** 1 Department of Occupational Health and Occupational Medicine, Guangdong Provincial Key Laboratory of Tropical Disease Research, School of Public Health, Southern Medical University, Guangzhou, China.; 2 Deparment of Biomedical Engineering, Faculty of Engineering, the Hong Kong Polytechnic University.; 3 Epithelial Cell Biology Research Centre, School of Biomedical Sciences, Faculty of Medicine, the Chinese University of Hong Kong.; 4 Department of Physiology, School of Medicine, Jinan University, Guangzhou, China; 5 Department of Pathology, The University of Hong Kong, Hong Kong, Hong Kong; 6 Department of Gynecology, Shenzhen Second People's Hospital, 518035 Shenzhen, China.; 7 Centre of Reproductive Medicine and Andrology, Shenzhen Second People's Hospital, 518035 Shenzhen, China.

**Keywords:** MRP4, Wnt/β-catenin, endometrium, embryo implantation, endometriosis, endometrial cancer.

## Abstract

**Rationale**: Abnormal Wnt/β-catenin signaling in the endometrium can lead to both embryo implantation failure and severe pathogenic changes of the endometrium such as endometrial cancer and endometriosis. However, how Wnt/β-catenin signaling is regulated in the endometrium remains elusive. We explored possible regulation of Wnt/β-catenin signaling by multi-drug resistance protein 4 (MRP4), a potential target in cancer chemotherapy, and investigated the mechanism.

**Methods**: Knockdown of MRP4 was performed in human endometrial cells *in vitro* or in a mouse embryo-implantation model *in vivo*. Immunoprecipitation, immunoblotting and immunofluorescence were used to assess protein interaction and stability. Wnt/β-catenin signaling was assessed by TOPflash reporter assay and quantitative PCR array. Normal and endometriotic human endometrial tissues were examined. Data from human microarray or RNAseq databases of more than 100 participants with endometriosis, endometrial cancer or IVF were analyzed. *In vitro* and *in vivo* tumorigenesis was performed.

**Results**: MRP4-knockdown, but not its transporter-function-inhibition, accelerates β-catenin degradation in human endometrial cells. MRP4 and β-catenin are co-localized and co-immunoprecipitated in mouse and human endometrium. MRP4-knockdown in mouse uterus reduces β-catenin levels, downregulates a series of Wnt/β-catenin target genes and impairs embryo implantation, which are all reversed by blocking β-catenin degradation. Analysis of human endometrial biopsy samples and available databases reveals significant and positive correlations of MRP4 with β-catenin and Wnt/β-catenin target genes in the receptive endometrium in IVF, ectopic endometriotic lesions and endometrial cancers. Knockdown of MRP4 also inhibits *in vitro* and *in vivo* endometrial tumorigenesis.

**Conclusion**: A previously undefined role of MRP4 in stabilizing β-catenin to sustain Wnt/β-catenin signaling in endometrial cells is revealed for both embryo implantation and endometrial disorders, suggesting MRP4 as a theranostic target for endometrial diseases associated with Wnt/β-catenin signaling abnormality.

## Introduction

The endometrium is a tissue layer of epithelial and stromal cells lining the uterus with its key physiological function in embedding the embryo/blastocyst for implantation 1-3. It undergoes tightly regulated changes throughout the menstruation cycle and is only receptive for implantation within a limited period of time 2, although such endometrial receptivity has not been fully understood. On the other hand, pathogenic changes of the endometrium can lead to severe diseases such as endometrial cancer and endometriosis, a painful disorder commonly seen in gynaecology clinics caused by migration and growth of endometrial tissues outside the uterine cavity 4, 5. However, mechanisms underlying the detrimental transformation of the endometrium have not been elucidated either.

The implanting embryos/placental cells are believed to share striking similarities with invasive cancer cells, and common pathways are activated in the endometrium during implantation and tumorigenesis 6. For instance, Wnt/β-catenin signaling has been observed to be activated in the endometrium upon the presence of the blastocyst in the uterus in mice 7-9. Conditionally ablation or overexpression of β-catenin in the mouse uterus results in fertility defects and abolishes decidualization 10, an endometrial stromal cell differentiation process required for embryo implantation 11. Aberrant activation of Wnt/β-catenin has also been reported in patients with endometriosis 12 or endometrial cancer 13. However, how Wnt/β-catenin signaling is regulated in the endometrium physiology or pathophysiology remains largely unexplored.

Multi-drug resistance protein 4 (MRP4), a member of the ATP-binding cassette (ABC) transporter subfamily C, is best known for its transporter function to exclude various drugs and therefore considered as a potential target to prevent drug-resistance, particularly in cancer chemotherapy 14-20. MRP4 is also known to transport endogenous signaling molecules such as prostaglandin E2 (PGE_2_). We have previously demonstrated that MRP4 is expressed in the endometrium and transports PGE_2_ 21, a key player in embryo implantation 11 22. Interestingly, PGE_2_ is reported to regulate Wnt/β-catenin signaling in other cell types 23, 24. We undertook the present study to investigate possible role of MRP4 in regulation of Wnt/β-catenin signaling in the endometrium and discovered unexpectedly that MRP4 acts in a manner independent of its PGE_2_-transporter function, interacting with and stabilizing β-catenin, to sustain the Wnt/β-catenin signaling for endometrial receptivity during embryo implantation, as well as in pathogenic transformation of the endometrium, i.e. endometriosis and endometrial cancer.

## Results

### MRP4 sustains Wnt/β-catenin signaling independent of transporter-function

**Figure 1:**
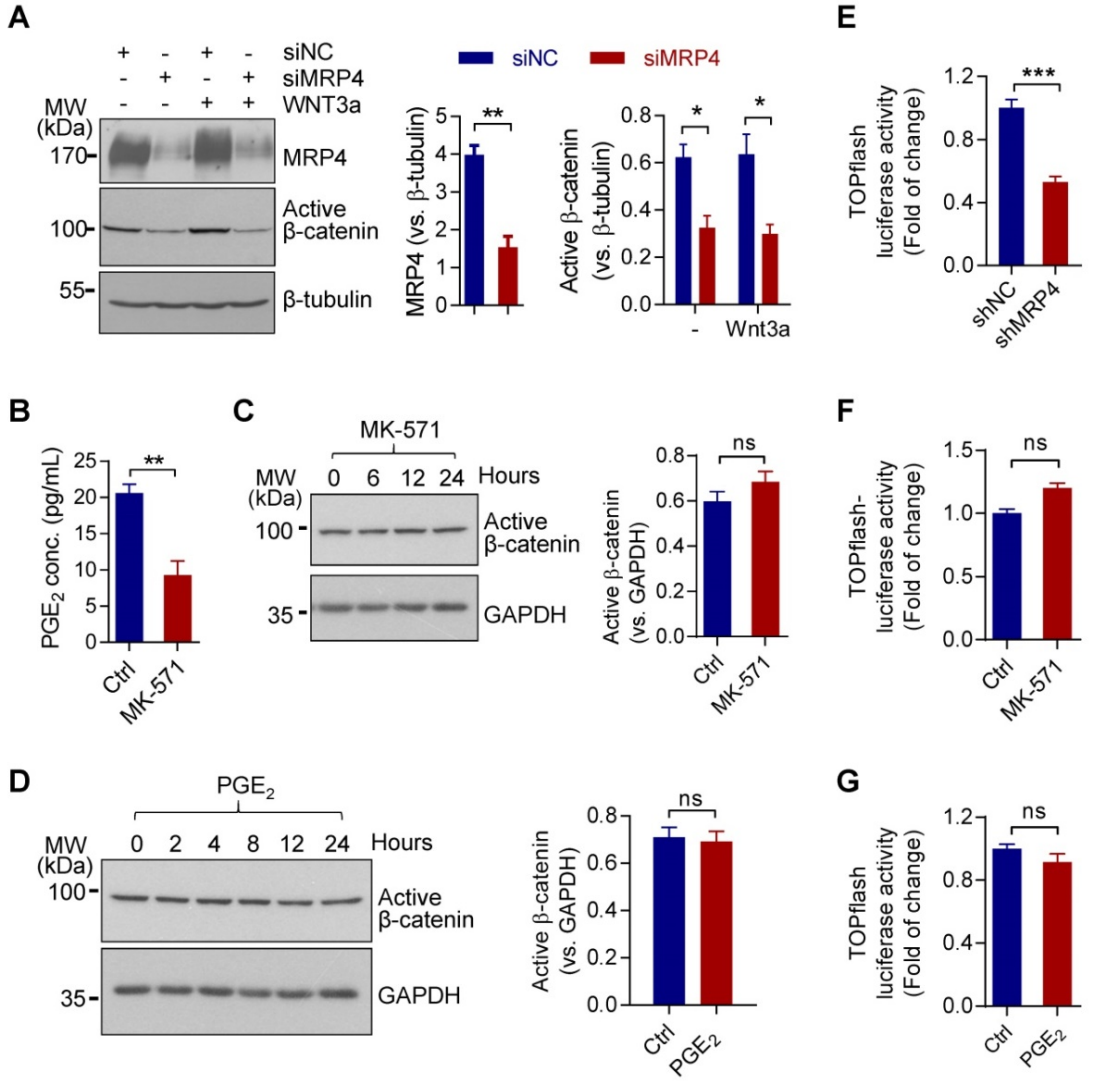
** MRP4 sustains Wnt/β-catenin signaling independent of transporting PGE_2_ in human endometrial epithelial cells. A)** Representative western blots with quantification for MRP4 and active β-catenin in human endometrial epithelial cells (ISK) treated with siRNAs against MRP4 (siMRP4) or non-silencing controls (siNC), in the presence (+) or absence (-) of Wnt3a (100 ng/ml). β-tubulin was used as the loading control. n = 6. *P < 0.05, **P < 0.01, by Student's *t* test. **B)** ELISA detection of PGE_2_ levels in culture medium of ISK cells incubated with or without (Ctrl) MK-571 (10 μM, a blocker of MRP4 transporter function). n = 4. **P < 0.01, by Student's *t* test. **C-D)** Representative western blots with quantification for active β-catenin in ISK cells incubated with MK-571 (10 µM, C) or PGE_2_ (10 µM, D) for 0-24 h. GAPDH was used as the loading control. n = 4. ns: not significant with *P* > 0.05, by Student's *t* test. **E-G)** Measurement of luciferase activity in TOPflash (β-catenin/TCF reporter)-transfected ISK cells treated with shRNAs (E, n = 3) against MRP4 (shMRP4) or non-silencing controls (shNC), or, incubated with or without (Ctrl) MK-571 (10 µM, F, n = 4) or PGE_2_ (10 µM, G, n = 4). ***P < 0.001, ns: not significant with *P* > 0.05, by Student's *t* test.

We first examined possible involvement of MRP4 in regulating Wnt/β-catenin signaling in a human endometrial epithelial cell line, Ishikawa (ISK). Knockdown of MRP4 by siRNAs (siMPR4) in ISK cells reduced levels of the active form of β-catenin (non-phosphorylated), as compared to cells treated with control siRNAs (siNC), in either the presence or absence of Wnt3a (100 ng/mL), a Wnt ligand reported to activate endometrial Wnt/β-catenin signaling 25 (Figure 1A). We further tested whether the effect of MRP4 on β-catenin activity was mediated by its PGE_2_-transporting function. To our surprise, despite successful blockage of PGE_2_ release (Figure 1B), the treatment with MK-571, the functional blocker of MRP4, for different time periods (10 μM, up to 24 h) did not affect the level of active β-catenin in ISK cells (Figure 1C). Similarly, treatment with exogenous PGE_2_ (10 μM, up to 24 h) did not affect the level of active β-catenin either (Figure 1D), excluding the involvement of PGE_2_ in mediating the effect of MRP4. To further confirm the effect of MRP4 on Wnt/β-catenin signaling, we transfected ISK cells with a Wnt/β-catenin signaling reporter, TOPflash luciferase, in conjunction with lenti-virus-packaged shRNAs targeting MRP4 (shMRP4). The results showed that compared to cells treated with control shRNAs (shNC), shMRP4-treated cells exhibited significantly decreased TOPflash luciferase activity (Figure 1E). However, treatment with MK-571 or exogenous PGE_2_ did not produce significant change in the TOPflash luciferase activity in ISK cells (Figure 1F&G). These results revealed an unexpected role of MRP4 in regulating Wnt/β-catenin signaling, which is independent of PGE_2_ or MRP4 transporter function.

### MRP4 interacts with and stabilizes β-catenin in human endometrial cells

**Figure 2:**
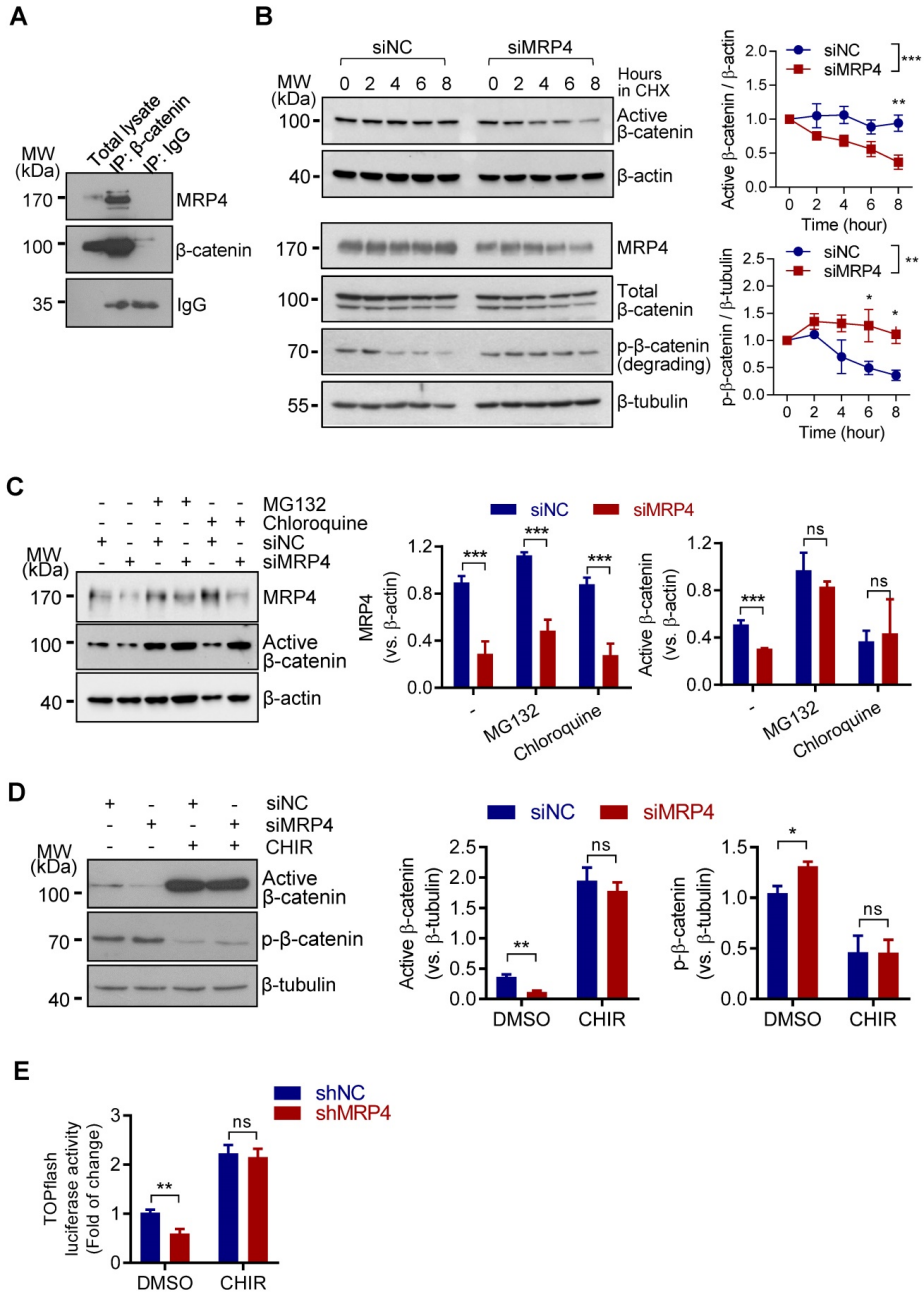
** MRP4 stabilizes β-catenin in human endometrial epithelial cells. A)** Representative western blots for MRP4 and β-catenin in ISK cells before (Total lysate) and after immuno-precipitated (IP) for β-catenin or IgG as the IP control. n = 3. **B)** Representative western blots with quantification for MRP4, active (non-phosphorylated), degrading (phosphorylated, p-β-catenin) and total (phosphorylated and non-phosphorylated) β-catenin in siNC- or siMPR4-transfected ISK cells incubated with cycloheximide (CHX, 10 μM, a protein synthesis inhibitor) for 0-8 h. β-actin or β-tubulin was used as the loading control. n = 3. *P < 0.05, **P < 0.01, ***P < 0.001, by two-way ANOVA. **C)** Representative western blots with quantification for MRP4 and active β-catenin in ISK cells transfected with siMRP4 or siNC, in the presence (+) or absence (-) of MG132 (10 μM, a proteasome inhibitor) or Chloroquine (10 μM, a lysosome inhibitor). β-actin was used as the loading control. n = 3. ***P < 0.001, ns: not significant with *P* > 0.05, by Student's *t* test. **D)** Representative western blots with quantification for active β-catenin and p-β-catenin in ISK cells transfected with siNC or siMRP4, in the presence (+) or absence (-) of CHIR-99021 (CHIR, 10 µM, an inhibitor of glycogen synthase kinase-3β blocking degradation-associated phosphorylation of β-catenin). β-tubulin was used as the loading control. n = 5. *P < 0.05, **P < 0.01, ns: not significant with *P* > 0.05, by Student's *t* test. **E)** Measurement of luciferase activity in TOPflash-transfected ISK cells treated with shMRP4 or shNC, in the presence of CHIR (10 µM) or DMSO as the control, n = 4. **P < 0.01, ns: not significant with *P* > 0.05, by Student's *t* test.

The observed effect of MRP4-knockdown, but not its transporter-functional inhibition, on β-catenin activity (Figure 1A-D) suggested that the presence of MRP4 protein *per ce* may affect the protein level of β-catenin in ISK cells. We suspected that such an effect of MRP4 on β-catenin might be mediated through protein-protein interaction, a well-documented mechanism to stabilize proteins, by preventing them from degradation in various cell types 26-30. Indeed, immunoprecipitation with an antibody against β-catenin successfully pulled down both β-catenin and MRP4 from protein extracts of ISK cells (Figure 2A). To confirm the effect of MRP4-knockdown on β-catenin protein stability, we treated ISK cells with cycloheximide (10 μM), a protein synthesis blocker, and found that the level of active β-catenin was rapidly decreased (by 63%) within 8 h in siMRP4-treated cells, significantly faster than that of siNC-treated cells (by 5%) (Figure 2B). On the other hand, with protein synthesis blockage for 8 h, the level of phosphorylated (degrading) β-catenin was reduced in control cells (siNC), whereas the siMRP4-treated cells showed sustained level of degrading β-catenin (Figure 2B), suggesting enhanced β-catenin degradation induced by MRP4-knockdown. Moreover, blocking protein degradation by incubating the cells with MG132 (a proteasome inhibitor, 10 μM) or chloroquine (a lysosome inhibitor, 10 μM) for 24 h successfully recovered the active β-catenin levels in siMRP4-treated cells (Figure 2C). To further confirm the role of MRP4 in preventing β-catenin degradation, we used CHIR99021 (CHIR), an inhibitor of glycogen synthase kinase 3 β (GSK3β), which blocks degradation-associated phosphorylation of β-catenin 31. After treating the cells with CHIR (10 μM) for 24 h, the phosphorylated β-catenin, indicating degradation, was largely abolished (Figure 2D), the active β-catenin level (Figure 2D) was effectively reversed and the Wnt/β-catenin TOPflash activity (Figure 2E) was recovered in cells with MRP4 knockdown (Figure 2E). These results in together suggested that MRP4, through protein-protein interaction, may stabilize β-catenin from degradation and thus sustain Wnt/β-catenin signaling in endometrial epithelial cells.

### MRP4 sustains Wnt/β-catenin signaling in the endometrium for embryo implantation in mice

**Figure 3:**
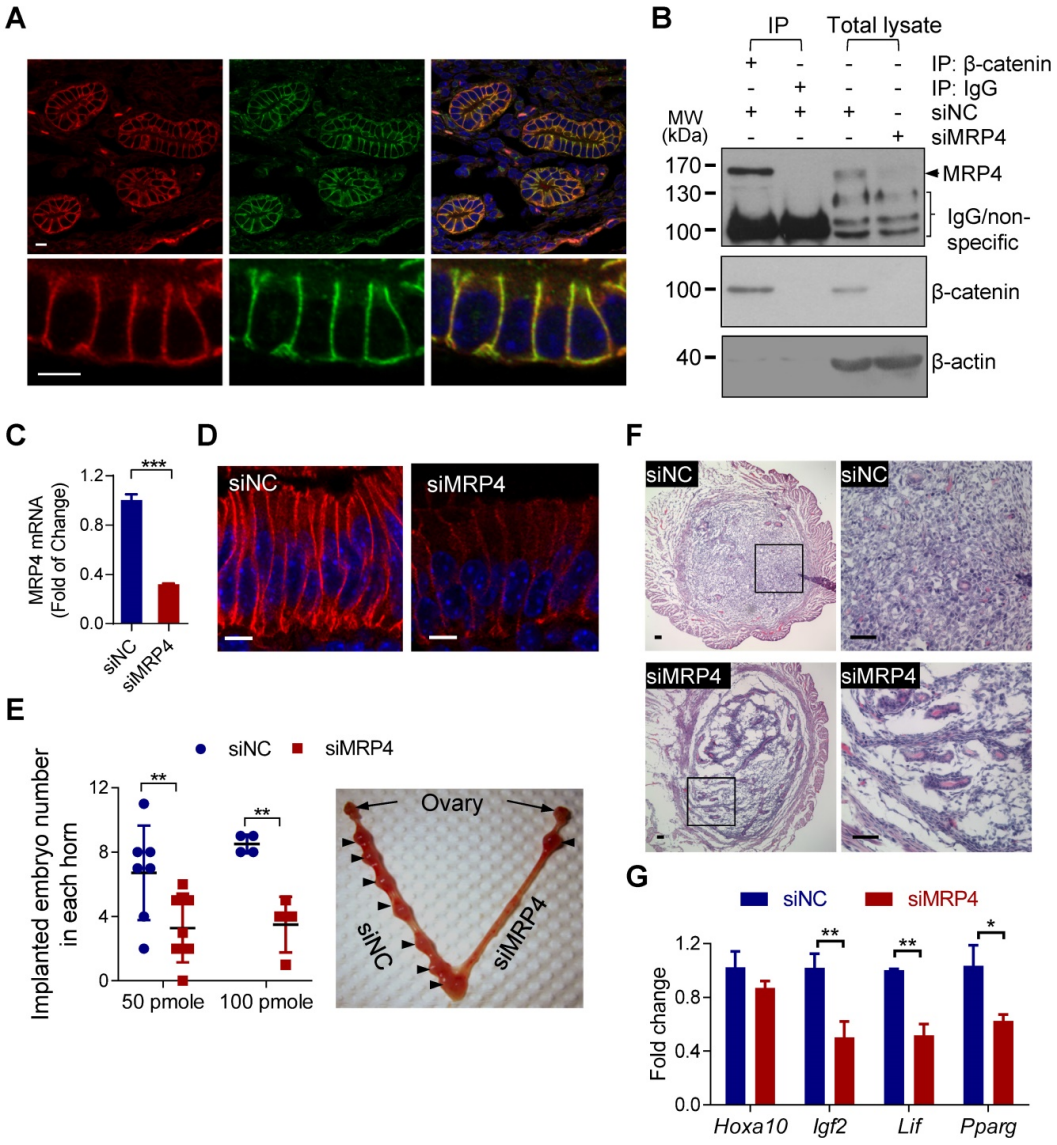
** MRP4 interacts with β-catenin in the endometrium required for embryo implantation in mice. A)** Confocal images of immunofluorescence labelling for MRP4 (red) and β-catenin (green) in mouse endometrium at 5 d.p.c. (days post coitum). Scale bars = 5 μm.** B)** Representative western blots for MRP4 and β-catenin in protein extracts from mouse uterine tissues (48 h after intrauterinally injected with siMRP4 or siNC, 100 pmole per uterine horn), before (Total lysate) and after immuno-precipitated (IP) for β-catenin or IgG as the IP control. n = 3.** C-D)** Quantitative PCR (qPCR) analysis (C, 4 d.p.c.) of and immunofluorescence staining (D, 5 d.p.c.) for MRP4 in mouse uteri treated with siMRP4 or siNC (100 pmole per uterine horn). n = 3. ***P < 0.001, by Student's *t* test. Scale bars in D = 5 µm. **E)** Implanted embryo numbers counted at 7 d.p.c. in uteri treated with siNC or siMRP4 (50-100 pmole per uterine horn). *Right*: Representative photograph of a mouse uterus injected with siMRP4 (100 pmole, right horn) or siNC (100 pmole, left horn) with arrows indicating implantation sites. n = 4-7. **P < 0.01, by Student's *t* test. **F-G)** Representative images of hematoxylin & eosin staining (F, 5 d.p.c, n = 4 mice) and qPCR analysis of *Hoxa10*,* Igf2, Lif*,* Pparg* (G, 5 d.p.c, n = 3-5) in mouse uteri treated with siMRP4 or siNC (100 pmole per uterine horn). *P < 0.05, **P < 0.01, by Student's *t* test. Scale bars in F = 50 μm.

We next asked whether MRP4 would interact with β-catenin *in vivo* and thus sustain the Wnt/β-catenin signaling in the endometrium required for embryo implantation. Double-labeling for β-catenin and MRP4 showed co-localization of these two proteins in endometrial epithelial cells in uterine tissues collected from pregnant mice at 5 d.p.c. (days post coitum), the day right after implantation takes place at mid-night of 4 d.p.c. (Figure 3A). Consistently, β-catenin and MRP4 can be pulled down together by immunoprecipitation from protein extracts of mouse uterus collected at 5 d.p.c. (Figure 3B), confirming protein-protein interaction between MRP4 and β-catenin in mouse endometrium at the period for embryo implantation.

We next performed *in vivo* knockdown of MRP4 in the uterus at implantation period by intrauterine injection with siRNAs against MRP4 (siMRP4, 50-100 pmole per uterine horn) at 3 d.p.c., shortly before implantation. Such operation led to successful knockdown of MRP4 at both mRNA (Figure 3C, 4 d.p.c.) and protein (Figure 3B&D, 5 d.p.c.) levels, and significantly reduced the number of implanted embryos at 7 d.p.c., as compared to the uteri treated with control siRNAs (siNC, Figure 3E). Histological analysis of uterine sections (Figure 3F, 5 d.p.c.) revealed that most cells in the siMRP4-treated endometrial stoma were loosely arranged and spindle-shaped, distinctively different from the massive amount of compactly arranged and round-shaped decidua cells seen in siNC-treated uteri, indicating defective decidualization, a prerequisite for embryo implantation. Moreover, siMRP4-treated uteri (Figure 3G, 5 d.p.c.) showed, as compared to siNC treated ones, lower expression levels of the genes essential to implantation, including *Igf2, Lif* and *Pparg*, which are also known to be regulated by Wnt/β-catenin signaling 32-34.

**Figure 4:**
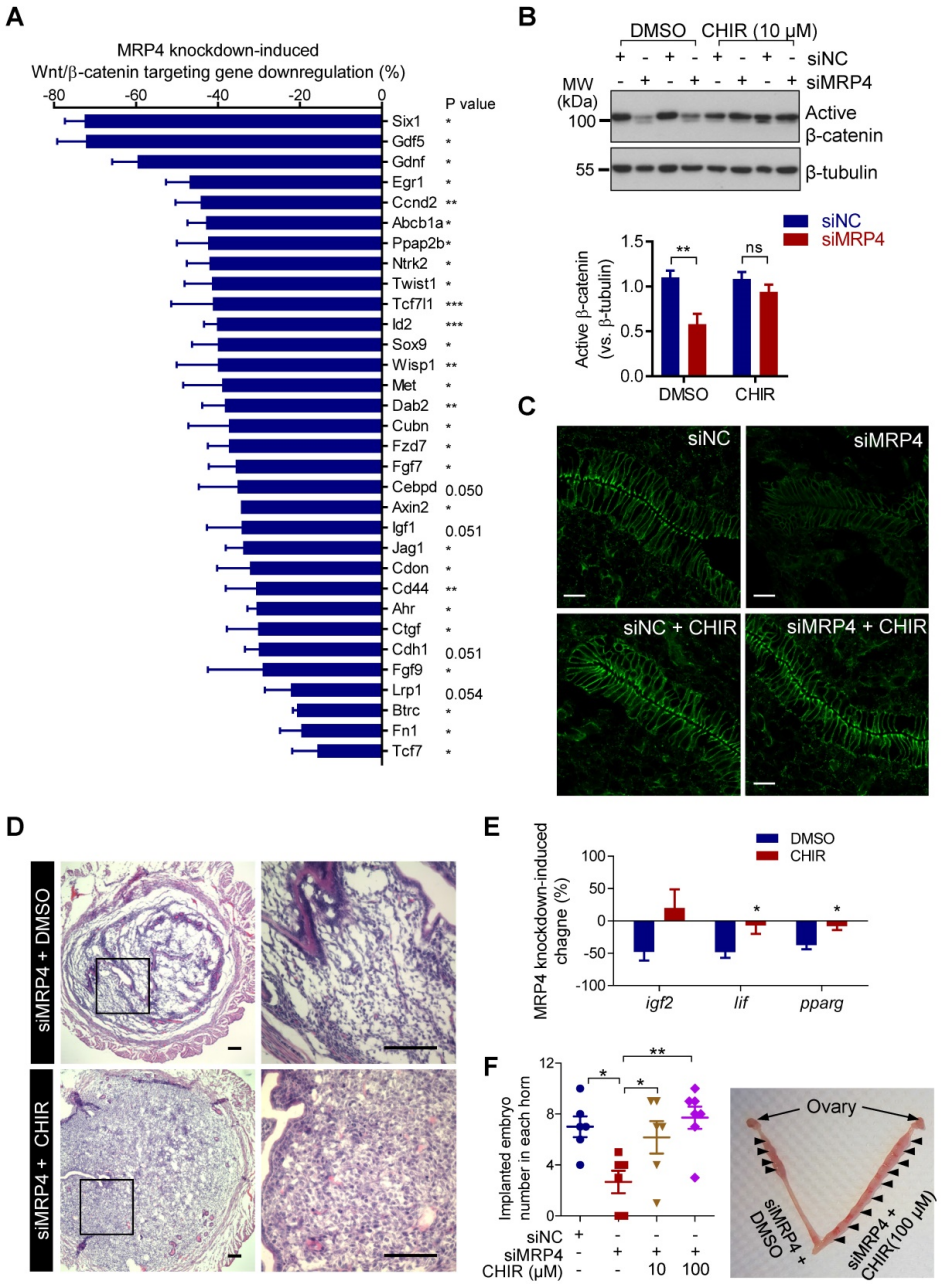
** MRP4 sustains Wnt/β-catenin signaling in the endometrium for embryo implantation in mice. A)** qPCR array showing downregulation of Wnt/β-catenin target genes in the mouse uterine horns (5 d.p.c) with MRP4 knockdown by siMRP4 (100 pmole per uterine horn), as compared to the paired uterine horns treated with siNC (100 pmole per uterine horn). Data are percentages of reduction in mRNA level of genes, (siMRP4-siNC) / siNC %. n = 3. *P* values are *(< 0.05), **(< 0.01), ***(< 0.001), or as indicated, by paired Student's *t* test. **B-C)** Representative western blots with quantification for active β-catenin (B) and immunofluorescence staining for total β-catenin (C) in mouse uteri (5 d.p.c.) injected with siNC or siMRP4 (100 pmole per uterine horn) and co-treated with CHIR (10 µM) or DMSO as the control. n = 3. **P < 0.01, ns: not significant with *P* > 0.05, by Student's *t* test. Scale bars in C = 10 µm. **D)** Representative images of hematoxylin & eosin staining in mouse uteri (5 d.p.c) with MRP4 knockdown (siMRP4) and treatment with DMSO or CHIR (10 µM). n = 4. Scale bars = 50 μm. **E)** qPCR analysis of *Igf2, Lif* and *Pparg* at 5 d.p.c. in mouse uteri injected with siNC or siMRP4 (100 pmole per uterine horn), and co-treated with CHIR (10 µM) or DMSO as the control. n = 3-5, *P < 0.05, by Student's *t* test. **F)** Implanted embryo numbers counted at 7 d.p.c. in mouse uteri injected with siNC or siMRP4 (100 pmole per uterine horn), and co-treated with CHIR (10-100 µM) or DMSO as the control. n = 6-7, *P < 0.05, **P < 0.01, by One-way ANOVA.

We next used a quantitative PCR array for Wnt/β-catenin signaling target genes to examine the mouse uterine tissues (5 d.p.c.) with MRP4 knockdown. 74 of total 84 genes in the array were successfully detected in the uterine tissues with most of them (61 genes) downregulated to different extend in siMRP4-treated uteri as compared to siNC-treated ones (Figure S1). Downregulation (siMRP4 versus siNC) of 28 genes (i.e.* Six1, Gdf5, Gdnf, Egr1, Ccnd2, Abcb1a, Ppap2b, Ntrk2, Twist1, Tcf7l1, Id2, Sox9, Wisp1, Met, Dab2, Cubn, Fzd7, Fgf7, Axin2, Jag1, Cdon, Cd44, Ahr, Ctgf, Fgf9, Btrc, Fn1 and Tcf7)* showed statistical significance (Figure 4A)*.* Consistently, significantly lower levels of active β-catenin (non-phosphorylated form, Figure 4B) and total β-catenin (Figure 4C) were found in the mouse uteri with MRP4 knockdown at 5 d.p.c., as compared to the uteri treated with siNC. These results suggested that the MRP4 knockdown-impaired Wnt/β-catenin signaling may underlie the observed implantation defect. To further prove this, we used CHIR to persistently activate Wnt/β-catenin signaling, in conjunction with the *in vivo* uterine MRP4 knockdown. The results showed that co-treatment with CHIR (10 μM, intrauterine injection) significantly boosted up the levels of active β-catenin (Figure 4B) and total β-catenin (Figure 4C). Moreover, CHIR improved decidualization (Figure 4D), recovered expression of implantation genes *Lif* and *Pparg* (Figure 4E) and, in a dose-dependent manner (10-100 μM), reversed the implantation rate in siMRP4-treated uteri (2.6 ± 0.9 per horn) back to normal levels (6.2 ± 1.3 per horn at 10 μM, and 7.7 ± 0.9 per horn at 100 μM, 7 d.p.c., Figure 4F), comparable to that of the siNC-treated group (7.0 ± 0.8 per horn). These results in together indicate a critical role of MRP4 in sustaining Wnt/β-catenin signaling in the endometrium for embryo implantation.

### MRP4 interacts and correlates with β-catenin in human endometrium

**Figure 5:**
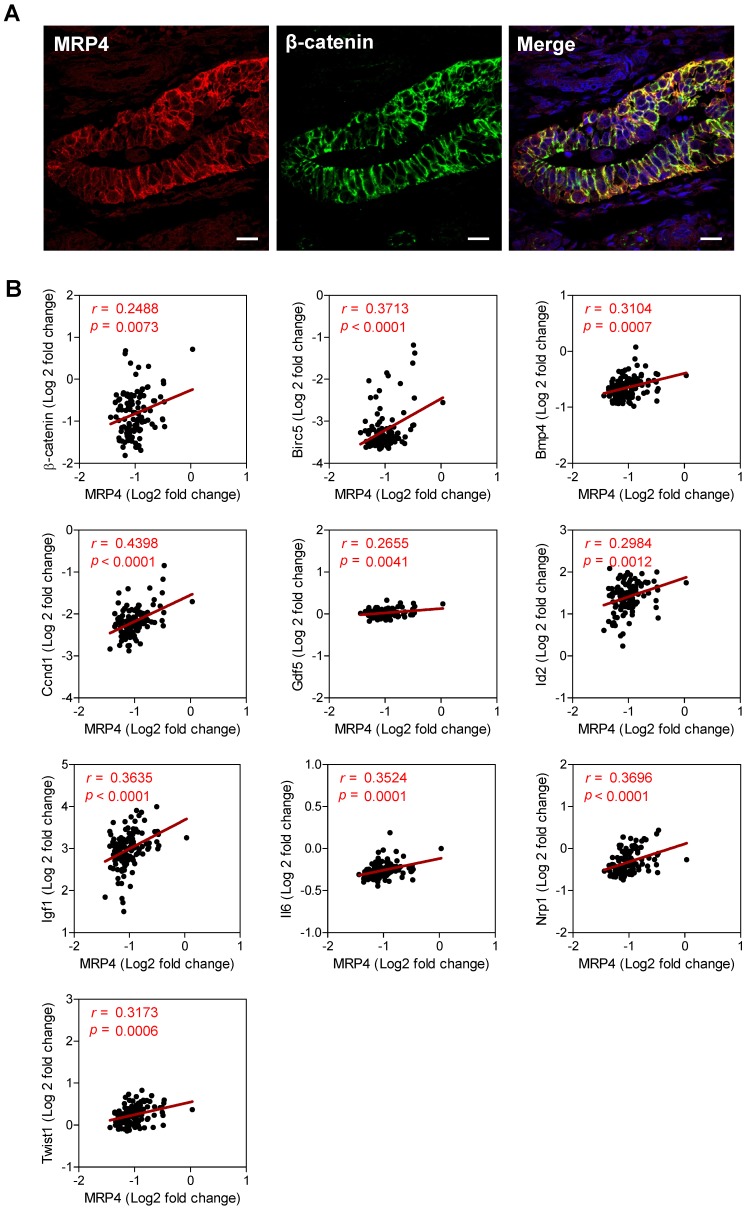
** MRP4 interacts with β-catenin and correlates with Wnt/β-catenin signaling genes in human endometrium. A)** Confocal images of immunofluorescence labelling for MRP4 (red) and β-catenin (green) in normal human endometrial tissues. Scale bars = 10 μm. **B)** Correlation analysis of mRNA levels of MRP4 and β-catenin or Wnt/β-catenin targeting genes (i.e. *Birc5, Bmp4, Ccnd1, Gdf5, Id2, Igf1, Il6, Nrp1* and* Twist1*) in human endometrial tissues collected at mid-secretory phase during IVF treatment (n = 115). Data were retrieved from a previously published dataset (GSE58144, http://www.ncbi.nlm.nih.gov/geo). Values of *r* and *P* are shown for each analysis by Pearson correlation test.

We next explored possible interaction/correlation between MRP4 and β-catenin in human endometrial tissues. Similar to that observed in mouse endometrium, immunostaining of MRP4 was found to be co-localized with that of β-catenin in normal human endometrial biopsy samples (Figure 5A). We also analyzed a previously published dataset from whole genome gene expression microarrays in endometrium tissues collected from women at mid-secretory phase (receptive window) during IVF treatment (n = 115) (accession number GSE58144) 35, which revealed significant and positive correlations of MRP4 with β-catenin (*r* = 0.2488, *p* = 0.0073) and Wnt/β-catenin target genes including *Birc5* (*r* = 0.3713, *p* < 0.0001)*, Bmp4* (*r* = 0.3104, *p* = 0.0007)*, Ccnd1* (*r* = 0.4398, *p* < 0.0001)*, Gdf5* (*r* = 0.2655, *p* = 0.0041)*, Id2* (*r* = 0.2984, *p* = 0.0012)*, Igf1* (*r* = 0.3635, *p* < 0.0001)*, Il6* (*r* = 0.3524, *p* = 0.0001)*, Nrp1* (*r* = 0.3696, *p* < 0.0001)* and Twist1* (*r* = 0.3173, *p* = 0.0006) (Figure 5B). These results suggest a consistent role of MRP4 in promoting Wnt/β-catenin signaling in receptive endometrium in humans as well.

### MRP4 correlation with Wnt/β-catenin signaling in human endometriosis and endometrium cancer

**Figure 6:**
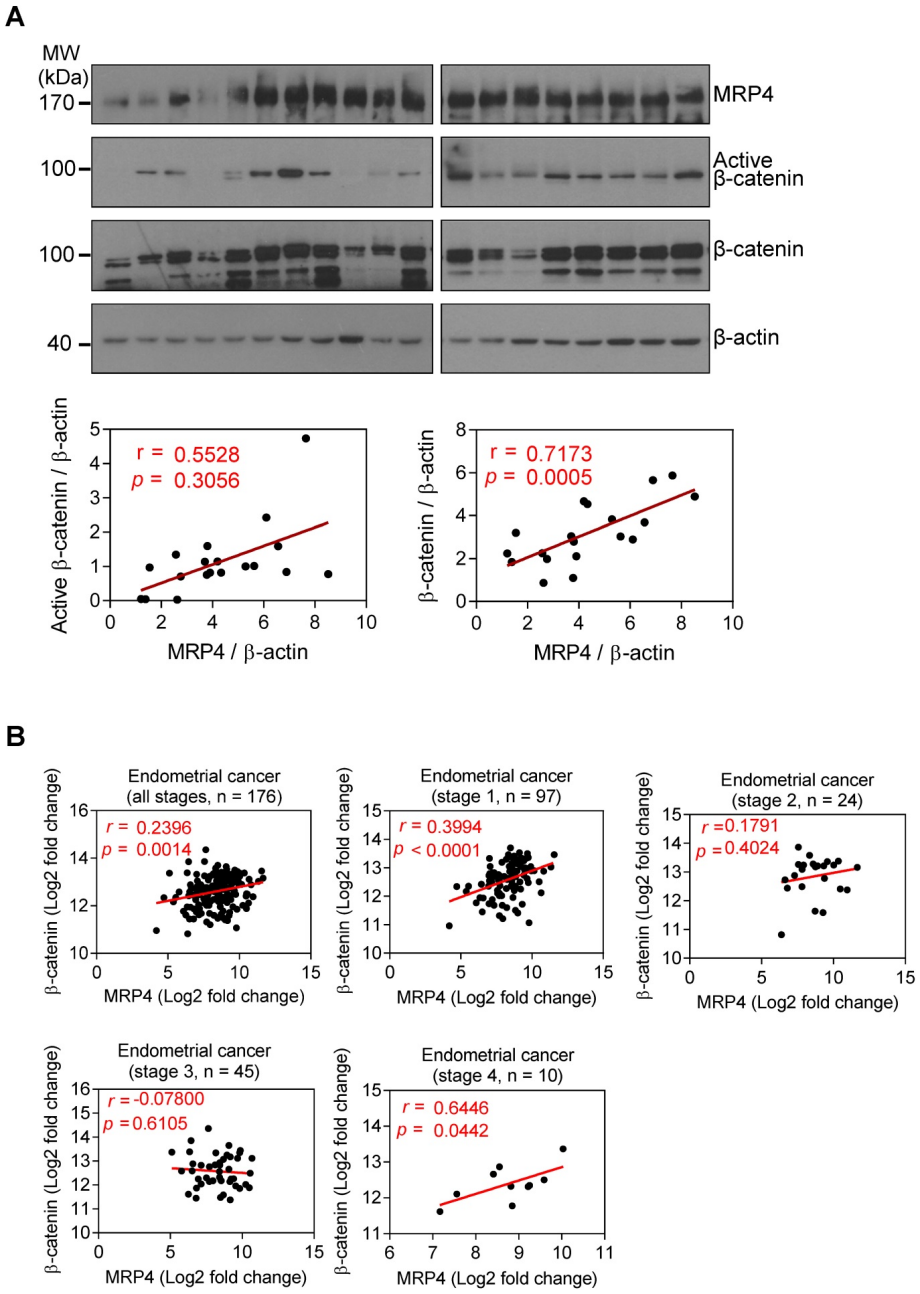
**MRP4 correlates with β-catenin in human endometriosis and endometrial cancer. A)** Western blots for MRP4, active β-catenin and β-catenin, with correlation tests of their quantified protein levels in human endometriotic samples (n = 19). β-actin was used as the loading control. Values of *r* and *P* are shown for each analysis by Pearson correlation test. **B)** Correlation analysis of MRP4 and β-catenin mRNA levels in human endometrial cancer (dataset ID: UCEC from TCGA Research Network: http://cancergenome.nih.gov/)

Since many of the Wnt/β-catenin target genes that were affected by MRP4-kockdown in the mouse uterus (Figure 4A) are associated with tumorigenesis (e.g. *Ccnd2*, *Twist1, Id2*, *Sox9*, *Wisp1, Met, Fzd7, Axin2, Jag1, CD44*, *Fgf9*, *and Fn1*) 36-47, we next examined possible correlation of MRP4 with Wnt/β-catenin signaling during detrimental transformation of the endometrium (e.g. endometriosis and endometrium cancer) in humans. Western blotting for MRP4 and β-catenin in ectopic endometrial lesions collected from total 19 women diagnosed of ovarian endometriosis (clinical data shown in Table 1) revealed a significant correlation of protein levels between MRP4 and total β-catenin (*r* = 0.7173, *p* = 0.0005) or active β-catenin (*r* = 0.5528, *p =* 0.0141) (Figure 6A). We also analysed an available human databases (TCGA Research Network: http://cancergenome.nih.gov/), and found significant correlations between MRP4 with β-catenin mRNA levels in endometrial cancers, in particular at stages I and IV (Figure 6B). We went further to explore possible correlation of MRP4 with β-catenin in other types of cancer and found significant correlations in colorectal, prostate, bladder and breast cancers (Figure S2). These results therefore suggest that in addition to the role in receptive endometrium, MRP4 may also be involved in regulating Wnt/β-catenin signaling in pathogenic endometrial transformation as well.

### MRP4 is involved in endometrial tumorigenesis *in vitro* and *in vivo*

**Figure 7:**
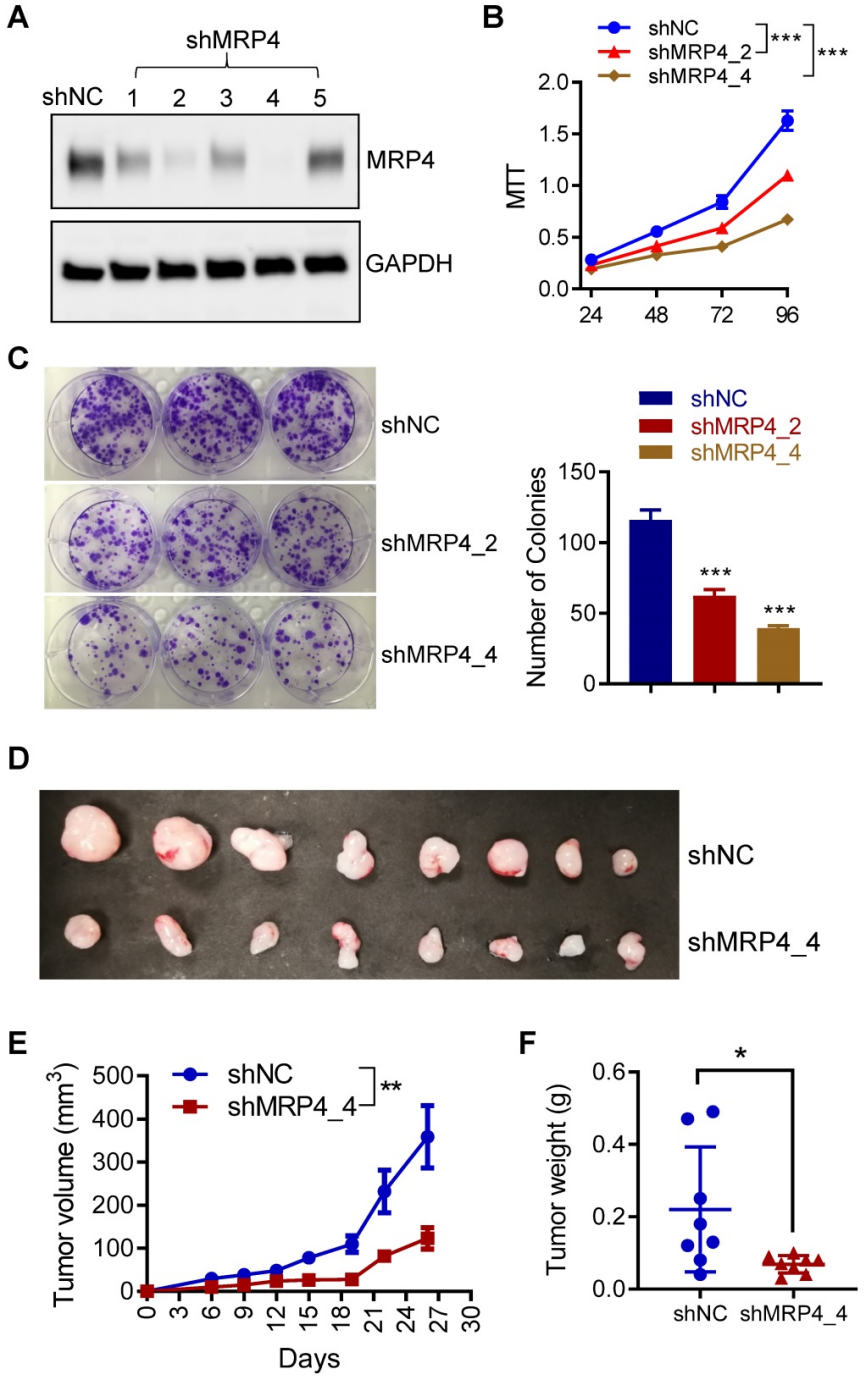
** MRP4 is involved in endometrial tumorigenesis *in vitro* and* in vivo*. A)** Western blots for MRP4 in ISK cells after stable expression of 5 designs of shRNAs against MRP4 (shMRP4_1, 2, 3, 4 and 5) or non-silencing controls (shNC). GAPDH was used as the loading control. **B-C)** MTT assay (B) and colony formation (C) in ISK cells with MRP4 knockdown by shMRP4_2 or shMRP4_4, or shNC controls. n = 3. *** P < 0.001 by two-way ANOVA in B and one-way AVOVA in C. **D-F)** Photographs of dissected tumors (D), measurement of tumor volume (E) and weight (F) after ISK cells treated with shNC or shMRP4_4 were subcutaneously inoculated into the flanks of nude mice (3 x 10^6^ cells per mouse) and grown for 26 days. n = 8. ** P < 0.01, by two-way ANOVA in E, * P < 0.05 by Student's *t* test in F.

To investigate the role of MRP4 in endometrial tumorigenesis, we next performed* in vitro* and *in vivo* tumorigenesis with ISK cells in conjunction of MRP4 knockdown by designed shRNAs. As shown in Figure 7A, two out of five designs of shRNAs against MRP4 (shMRP4_2 and shMRP4_4) gave high knockdown efficiency and were therefore used in the following experiments. Such knockdown of MRP4 (by shMRP4_2 or shMRP4_4) resulted in significantly reduced proliferation (Figure 7B) and colony formation (Figure 7C) in ISK cells, as compared to cells treated with negative control shRNAs (shNC). We also subcutaneously injected ISK cells with MRP4-knockdown (by shMPR4_4) in nude mice. The growth of ISK xenografts in nude mice during 26 days after transplantation was significantly slower in the MRP4-knockdown group (shMRP4), as compared to the controls (shNC, Figure 7D and Figure 7E). Both tumour volume (Figure 7E) and weight (Figure 7F) were significantly reduced in the MRP4-knockdown group as compared to the controls (shNC) at the end (day 26). These results therefore confirmed a role of MRP4 in endometrial tumorigenesis.

## Discussion

**Figure 8:**
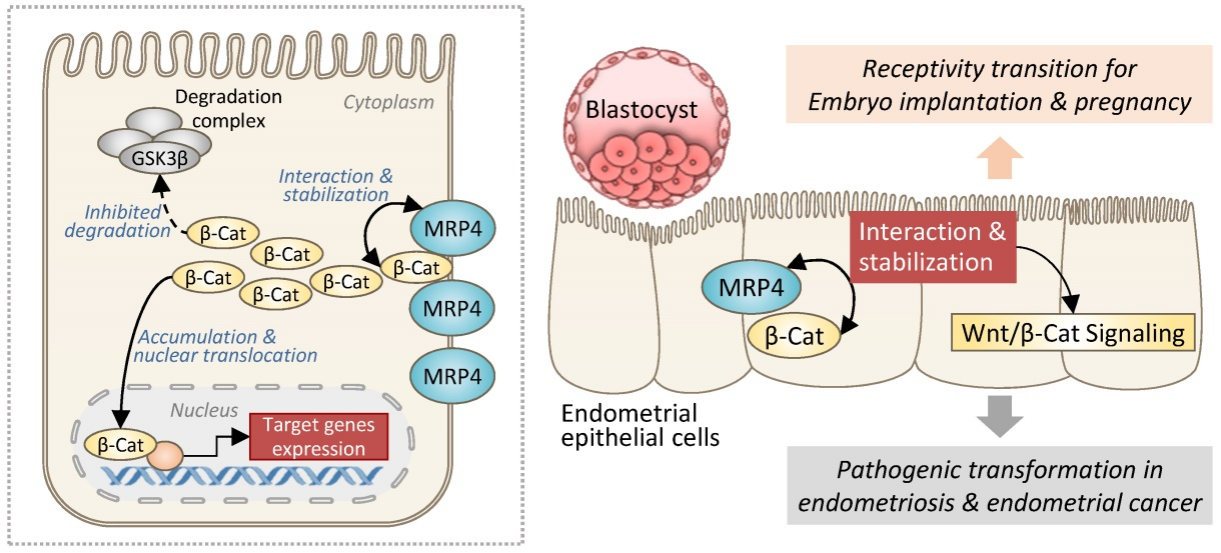
**Schematic drawing showing the role of MRP4 in regulation of Wnt/β-catenin signaling in the endometrium.** In endometrial epithelial cells, MRP4, through protein-protein interaction, stabilizes β-catenin (β-Cat) from degradation, which accumulates sufficient amount of β-catenin to translocate into the nucleus leading to the transcription of target genes of Wnt/β-catenin signaling pathway. Such a role of MRP4 in the endometrium is involved in both receptivity transition for embryo implantation and pathogenic transformation to develop endometriosis or endometrial cancer.

Taken together, the present study has revealed a role of MRP4 in sustaining Wnt/β-catenin signaling in the endometrium through a previously unidentified mechanism, which is independent of its transporter function but instead via interacting with and stabilizing β-catenin (Figure 8). Such a role of MRP4 in the endometrium is involved in both receptivity transition for embryo implantation and pathogenic transformation to develop endometriosis or endometrial cancer (Figure 8).

The present observation of impaired decidualization and embryo implantation rate in the MRP4 knockdown mice suggests that MRP4 is required for adequate endometrial receptivity for a successful pregnancy, which is consistent with previously reported smaller litter size in MRP4 knockout mice 48. We have previously reported that blocking MRP4's PGE_2_-transport function also inhibits embryo implantation in mice 21. In light of the present finding, it appears that the involvement of MRP4 in embryo implantation may be two-fold. First, it provides the mechanism for directional transport of PGE_2_ to stroma, a key event necessary for inducing stromal decidualization for embryo implantation 11. Second and unexpectedly, MRP4 acts to sustain the Wnt/β-catenin signaling for embryo implantation, in a PGE_2_-independent manner. Although PGE_2_ has been reported to regulate Wnt/β-catenin signaling in other cell types, it does not seem to exert detectable effect on the Wnt/β-catenin signaling in the endometrial epithelial cells. The importance of MRP4-dependent Wnt/β-catenin signaling for embryo implantation is evidenced by the fact that the retrieval of active β-catenin levels by blocking β-catenin degradation can rescue the MRP4 knockdown-induced implantation defects. This is further supported by the significant correlation between MRP4 and β-catenin, as well as MRP4 and Wnt/β-catenin downstream target genes, in human endometrial biopsy samples collected at mid-secretary phase during IVF treatment.

The present study has demonstrated a previously unsuspected protein-protein interaction between MRP4 and β-catenin, which is shown to be important for sustaining the level of active β-catenin in the endometrium. The level of β-catenin inside the cell is a key to the canonical Wnt/β-catenin signaling 49. For a successful embryo implantation, endometrial level of β-catenin seems to be crucial since manipulation β-catenin expression, since either depletion or overexpression of β-catenin, in the endometrium, resulted in implantation failure in mice 10. Of note, protein-protein interactions have been well documented to improve protein stability by preventing them from degradation in various cell types 26-29. The interaction between β-catenin and CFTR, another ABCC family member, has previously been demonstrated to stabilize β-catenin essential to various physiology or pathophysiology events such as embryonic development 27 and intestinal inflammation 26.

In the present study, an enhanced stability of β-catenin by MRP4 is also observed as evidenced by 1) more rapid degradation of β-catenin (increase in degrading form and decrease in active form) in MRP4-knockdown cells; and 2) reversed β-catenin protein level by blocking either protein degradation in general (MG132 or chloroquine) or β-catenin degradation specifically (CHIR). Therefore, the observed interaction of MRP4 with β-catenin may contribute to the stability of β-catenin and thus underlie the capacity of MRP4 in sustaining Wnt/β-catenin signaling.

Apart from its demonstrated role in endometrial receptivity, the presently discovered MRP4-dependent Wnt/β-catenin signaling appears to be involved in pathological transformation of the endometrium, i.e. endometriosis and endometrial cancer, as well. A correlation of MRP4 with β-catenin is consistently found in clinical samples of endometriosis and endometrial cancer, as demonstrated in the present study or from big databases. Although databases are limited to mRNA level analysis, interaction and correlation between MRP4 and β-catenin proteins in cancer tissues are still possible. Importantly, *in vitro* and *in vivo* results confirms a role of MRP4 in endometrial tumorigenesis. While both the expression of MRP4 and the involvement of Wnt/β-catenin signaling have been reported in endometriosis and endometrial cancer 50-52, a possible link between the two has never been suspected. Present findings suggest correlation of MRP4 with Wnt/β-catenin signaling in the endometrium both at embryo implantation and in pathogenic transformation. This is consistent with the general recognition that embryo implantation and tumorigenesis may share common pathways 6, although the difference between the two processes in MRP4 or Wnt/β-catenin signaling awaits further investigation. Interestingly, the analyses of databases for cancers from other tissues, such as colon, prostate, bladder and breast, also show significant correlations between MRP4 and β-catenin, indicating the possible involvement of the MRP4-dependent Wnt/β-catenin signaling in a wide spectrum of physiological or pathological processes in different tissues.

The non-transporter role of MRP4 discovered in the present study raises an interesting possibility that ABC transporters might play an important role in signal transduction independent of their transporter function. The roles of ABC transporters expressed in different tissues may have to be re-examined in light of the present finding. How MRP4 or multi-drug resistance family proteins should be targeted/inhibited in cancer chemotherapy needs to be re-considered, since simple transport-blockage may not be effective to attenuate Wnt/β-catenin signaling, a key pathway activated in tumorigenesis. Moreover, given the versatile roles of Wnt/β-catenin signaling in a variety of different cellular processes and the wide distribution of MRP4, as well as other ABC transporters, the presently discovered novel role of MRP4 in the regulation of Wnt/β-catenin signaling may have far-reaching implications beyond embryo implantation and tumorigenesis.

## Methods

### Mice and intrauterine injection

Institute of Cancer Research (ICR) mice were obtained from the Laboratory Animal Service Centre of the Chinese University of Hong Kong. All animal experiments were conducted in accordance with the university guidelines on animal experimentation, and approval by the Animal Ethics Committee of the Chinese University of Hong Kong was obtained for all related procedures. The day a vaginal plug was found after mating was identified as 1 d.p.c. The intrauterine injection surgery under general anesthesia was performed on 3 d.p.c. after mating as previously described 11. Dorsal midline skin incision was made and followed by two small incisions into the muscle wall near each ovary to expose the uterine-oviduct connecting region. Reagents were injected at the uterus-oviduct junction toward the uterine lumen. Afterwards, wounds were closed by suture and the mice were placed on a 37 ^o^C warmer till woken up from the anesthesia. The mice were closely monitored for 1 to 4 consecutive days after surgery. Mice were sacrificed by CO_2_ asphyxiation on 4, 5 and 7 d.p.c.

### Cell culture

The human endometrial epithelial cell line (adenocarcinoma line), Ishikawa (ISK), was purchased from ATCC (Viginia, United States) and cultured in RPMI-1640 supplemented with 10% fetal bovine serum (v/v) and 1% penicillin-streptomycin (v/v) in 5% CO_2_ incubators at 37 ^o^C. The cell line was recently authenticated by STR profiling at the Department of Anatomical and Cellular Pathology, Faculty of Medicine, The Chinese University of Hong Kong 53.

### RNA extraction, reverse transcription and quantitative PCR

Total RNA of cells or uterine tissues were extracted using TRIzol reagent (Invitrogen Life Technologies) according to manufacturer's instructions. 1 μg total RNA was used for reverse transcription reaction using M-MLV reverse transcriptase (Promega) according to the manufacturer's instructions. SYBR green assay was used for mouse genes: MRP4 (Primers: *forward* 5'-TCCCTTGTTCTGGCGAAGAC-3' and *reverse* 5'-CGAAGACGATGACTCCCTCG-3'), HoxA10 (Primers: *forward* 5'-AATGTCATGCTCGGAGAGCC-3' and *reverse* 5'-CTTCATTACGCTTGCTGCCC-3'), Igf2 (Primers: *forward* 5'-GTACAATATCTGGCCCGCCC-3' and *reverse* 5'-GGGTATGCAAACCGAACAGC-3'), Lif (Primers: *forward* 5'-GCCCAACAACGATGGTGTCA-3' and *reverse* 5'-CCCGTGTTTCCAGTGCAGA-3'), Pparg (Primers: *forward* 5'-GTCACACTCTGACAGGAGCC-3' and *reverse* 5'-ATCACGGAGAGGTCCACAGA-3') and Gapdh (Primers: *forward* 5'-TCTCCTGCGACTTCAACAGC-3' and *reverse* 5'-AGTTGGGATAGGGCCTCTCTT-3'). Quantitative PCR with SYBR Green Master Mix (Tli RNase H Plus, Takara) was carried out in triplicate on a 96-well plate using a QuantStudio 7 Flex Real-time PCR system (Applied Biosystems). Gapdh was used as an internal control for normalization and data were calculated using the 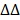
C_T_ method.

### Quantitative PCR array

1 µg total RNA extracted from each mouse uterine tissue sample was used for reserve transcription by RT^2^ First Strand Kit (Qiagen), and followed with the RT^2^ Profiler PCR array for Wnt signaling target genes (SABiosciences array PAMM-243Z), according to manufacturer's guide. The array was performed using SYBR Green PCR Master Mix (Applied Biosystems) in QuantStudio 7 Flex Real-time PCR system (Applied Biosystems). Data were analyzed using the online SABioscience Array data analysis software.

### Protein stability/degradation assay

ISK cells with or without MRP4 knockdown were incubated with cycloheximide (10 μM), a protein synthesis blocker, for 0, 2, 4, 6 or 8 h before proteins were extracted and analyzed by immunoblotting. In some experiments, cells were treated with MG132 (a proteasome inhibitor, 10 μM) or chloroquine (a lysosome inhibitor, 10 μM) or CHIR99021 (CHIR, 10 μM), an inhibitor of glycogen synthase kinase 3 β (GSK3β), which blocks degradation of β-catenin.

### Immunoblotting

The cells or tissues were lysed in ice-cold RIPA lysis buffer (50 mM Tris-Cl, pH 7.5, 150 mM NaCl, 1% NP-40, 0.5% DOC and 0.1% SDS) with protease and phosphatase inhibitor cocktail (catalog #78443, Thermo Scientific) for 30 min on ice. Supernatant was collected after centrifugation at 13,000 rpm for 30 min. Equal amounts of protein were resolved by SDS-polyacrylamide gel electrophoresis and electro-blotted onto equilibrated nitrocellulose membrane. After blocking in Tris-buffered saline (TBS) containing 5% non-fat milk, the membranes were immunoblotted with primary antibody of target proteins overnight at 4^ o^C. Antibodies against MRP4 (1:100, Abcam, ab15602); Non-phospho (Active) β-catenin (1:1000, Cell Signaling, 8814), β-catenin (1:1000, Cell Signaling, 9562), p-β-catenin (1:1000, Cell Signaling, 9561), β-tubulin (1:2000, Santa Cruz, sc-9104) and β-actin (1:5000, Sigma, A1978) were used. After three washes in TBS containing 0.1% Tween 20 (TBST), membranes were further incubated with HRP-conjugated antibodies and visualized by the enhanced chemiluminescence assay (GE Healthcare, UK) following manufacturer's instructions. Densitometry of Western blots was performed by Image J software.

### Immunoprecipitation

Cells were lysed with 1 ml of ice-cold lysis buffer (50 mM HEPES, 420 mM KCl, 0.1% NP-40 and 1 mM EDTA) with protease and phosphatase inhibitor cocktail for 30 min to 1 h on ice. After centrifugation at 13,000 rpm for 30 min at 4 ^o^C, supernatants were collected as protein extracts, which were re-suspended in 1 ml immunoprecipitation binding buffer (50 mM Tris, pH 7.5, 150 mM NaCl) with protease and phosphatase inhibitor cocktail and incubated with primary anti-β-catenin (1:50, Cell Signaling, 2677) or mouse IgG (1:100, Santa Cruz, sc-2025) as the control in conjunction with protein A/G beads (GE Healthcare) on rotator overnight at 4 ^o^C. The protein-antibody-bead complexes were washed three times with binding buffer using a magnetic separator. In the end, proteins complexes were eluted from the beads by SDS sample buffer (Invitrogen) with 4% β-mercaptoethanol, and further analyzed by immunoblotting.

### Immunofluorescence

Uterus tissues were harvested and fixed by immersion in 4% paraformaldehyde overnight at 4 ^o^C. After three times washed in PBS, uterine tissues were cryoprotected in 30% sucrose in PBS at 4 ^o^C for 24 h, mounted in OCT embedding media (Tissue-Tek, 4583, Sakura), and cryo-sectioned into 5 μm sections. Sections were rehydrated in PBS for 5 mins and microwaved in citrate buffer (pH 6.0) for 20 mins to retrieve antigens. After cooled down to room temperature and treated with 1% SDS in PBS for 4 mins, sections were blocked with 1% bovine serum albumin in PBS for 15 mins, incubated with primary antibody (MRP4, 1:20, Abcam, ab15602) and/or β-catenin (1:100, Cell Signaling, 2677) overnight at 4 ^o^C and subsequently fluorochrome-conjugated secondary antibody (invitrogen) for 1 h at room temperature. DAPI was used to stain cell nuclei. Images were acquired with a confocal microscope (Zeiss, Germany).

### RNA interference and gene knockdown

siRNAs against MRP4 (siMRP4, Cat#1299001, Assay-ID HSS115675, Invitrogen; 5'-GAGAAAGAAGGAGAUUUCCAAGAUU-3') and a negative control Med GC Duplex (siNC, Cat#12935300, Invitrogen) were used for knockdown of MRP4. For in vivo knockdown, 50-100 pmole siRNAs together with Lipofectamine 2000 (5 μl) in 10 μl Opti-MEM were injected into each uterine horn. In cells, 100 nM siRNAs were transfected with Lipofectamine 2000. Lenti-virus (LV3) packaged shRNAs targeting human MRP4 (shMRP4_1, 5'-GAGAAAGAAGGAGATTTCCAAG-3') and scrambled non-coding shRNAs (shNC, 5'-TTCTCCGAACGTGTCACGTTTC-3') were purchased from GenePharma (China). The viruses (2
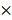
10^7^ TU/ml) were infected into ISK cells with Polybrene (5 μg/ml). The cells were cultured for three passages with puromycin (4 µg/ml) and selected stable clones for further experiments. For tumorigenesis assays, additional 4 designs of shRNAs (shMRP4_2: 5'-CCACCAGTTAAATGCCGTCTA-3', shMRP4_3: 5'-GCTCCGGTATTATTCTTTGAT-3', shMRP4_4: 5'-GCCTTCTTTAACAAGAGCAAT-3' and shMRP4_5: 5'-GCCTTACAAGAGGTACAACTT-3') together with the shMRP4_1 were purchased. To package the shRNAs into lentivirus, envelope vector pMD2.G (12259, Addgene) and packaging vectors psPAX2 (12260, Addgene) together with the shRNAs were transfected into 293T cells using lipofectamine 2000 (Invitrogen). Packaged virus was harvested 72 h afterwards and used for next transfection into ISK cells for 48 h. Virus-transfected ISK cells were grown in the presence of puromycin (4 µg/ml) for 14 days to select cells with stable expression of shRNAs.

### Luciferase assay

Cells were transiently transfected with TOPflash reporter gene (TCF reporter plasmid, 21-170, Millipore) and Renilla-Luc as an internal control, respectively. Cells were harvested 48 h after transfection in passive lysis buffer (Promega) and assayed by the Dual Luciferase Reporter Assay System (Promega) using GloMax luminometer (Promega). Renilla luciferase activity was used for normalization.

### PGE_2_ ELISA

ISK cells were incubated with 1 % FBS in DMEM culture medium for 8 h to synchronize the cells before FBS-free DMEM medium was used for all the treatments. Cell-free supernatant with PGE_2_ content was collected and measured using an EIA kit (Cayman Chemical, 514010).

### Human tissue collection

Women at the Second People's Hospital of Shenzhen diagnosed of ovarian endometriosis were recruited for the study. Tissues were collected at the proliferative phase from walls of the ovarian endometriomas during gynecological laparoscopic surgery. All samples were collected with informed consent from each woman and approval by the Institutional Review Board of the Second People's Hospital of Shenzhen (Ethics Approval No. 201306015). Individuals receiving hormone therapy or anti-inflammatory agents were excluded. Clinical data are shown in Table 1.

### MTT assay

Cells were seeded in 96-well plates at a density of 2000 cells per well and incubated for 1-4 days at 37 °C. Directly after removal of the medium, 200 μl of MTT (0.5 mg/mL, Sigma-Aldrich) was added in each well for incubation at 37 °C for 4 h. The medium and MTT solution were removed from each well and formazan crystals were dissolved in 100 μl of DMSO before the absorbance was measured at 570 nm using a plate spectrophotometer.

### Colony formation assay

Cells were seeded into 6-well plates at a density of 500 cells per well. The growth medium was replaced with fresh ones every 2 days. After culturing for 10 days, cells were fixed with methanol and stained with crystal violet. Colonies comprising > 50 cells were counted.

### In vivo tumorigenicity

Ncr-nu/nu-nude mice were provided by Laboratory Animal Unite, the University of Hong Kong. The protocols were performed after approval by the Animal Ethics Committee, the University of Hong Kong according to issued guidelines. 3 × 10^6^ of cells mixed with an equal volume of matrigel (BD Pharmingen) were injected subcutaneously at the back of 6-week-old female Ncr-nu/nu-nude mice. Tumor sizes were monitored every 3 days using digital vernier calipers, and tumor volumes were calculated using the formula [sagittal dimension (mm) × cross dimension (mm)^2^]/2 and expressed in mm^3^.

### Data availability

Human gene expression datasets of IVF endometrium (accession number: GSE58144), colorectal cancer (GSE24551, GSE24549 and GSE75315), prostate cancer (GSE46691 and GSE21034), bladder cancer (GSE57933) and breast cancer (GSE12093) were retrieved from the Gene Expression Omnibus (GEO, http://www.ncbi.nlm.nih.gov/geo). Datasets of endometrial carcinoma (ID: UCEC) and colon carcinoma (ID: COAD) were retrieved from The Cancer Genome Atlas (TCGA, http://cancergenome.nih.gov/). Data from normal or undetermined biopsy samples were excluded for analyzing cancer datasets. R2 platform (http://r2.amc.nl) was used for data retrieval and analysis. Correlation analysis was performed using Log2 transformed microarray data.

### Statistical analysis

The software GraphPad Prism 6.0 was used for graphing and statistical analyzing the data. Data are shown as mean ± SEM. Student's paired or unpaired t-test was used for two-group comparison. One-way or Two-way ANOVA was used for comparing three or more groups. Pearson test was used for correlation analysis. P < 0.05 was considered as statistically significant. The variance was calculated to be similar between comparison groups or otherwise corrected for particular tests.

## Supplementary Material

Supplementary figures.

## Figures and Tables

**Table 1:** Clinical data of subjects diagnosed of ovarian endometriosis

Subject#	Age	Endometriosis Stage	Affected ovary (Unilateral or bilateral)	Number of gravidity (G) and parity (P)	Chronic pelvic pain (No/mild/medium/severe)
1	25	III	Unilateral/Left	G0P0	Severe
2	27	Undetermined	Unilateral/Left	G0P0	Medium
3	25	II	Unilateral/Left	G0P0	Medium
4	25	IV	Unilateral/Right	G0P0	Severe
5	42	II	Unilateral/Left	G3P1	Mild
6	34	II	Unilateral/Right	G0P0	Mild
7	32	III	Unilateral/Right	G1P0	Medium
8	39	III	Unilateral/Right	G1P0	Mild
9	31	III	Bilateral	G0P0	No
10	25	III	Unilateral/Left	G0P0	No
11	27	II	Unilateral/Left	G1P0	No
12	27	III	Unilateral/Left	G1P0	Medium
13	29	III	Unilateral/Right	G0P0	Medium
14	30	III	Bilateral	G0P0	No
15	41	III	Unilateral/Left	G3P0	No
16	34	II	Bilateral	G2P0	No
17	29	IV	Unilateral/Left	G1P0	No
18	35	III	Unilateral/Left	G1P0	No
19	42	III	Unilateral/Right	G2P1	No
