# Supplementary material for: MRP4 sustains Wnt/β-catenin signaling for pregnancy, endometriosis and endometrial cancer
Source: Theranostics. 2019 Jul 9;9(17):5049–64. doi: 10.7150/thno.32097 (PMC6691374; doi:10.7150/thno.32097)

Supplementary Figures

Supplementary Figure 1. Effect of MRP4 knockdown on Wnt/β-catenin target genes in mouse uterus during embryo implantation.

A) Heat map of data from a qPCR array for Wnt/β-catenin target genes in the mouse uterine horns (5 d.p.c.) with MRP4 knockdown by siMRP4 (100 pmole per uterine horn), as compared to the paired uterine horns treated with siNC (100 pmole per uterine horn) as the control. Data are percentages of reduction in the mRNA level of genes, (siMRP4-siNC) / siNC %. n = 3. Blue to red color indicates downregulation to upregulation.

B) Volcano plot of fold-changes (siMRP4/siNC) and *P* values (by paired *t*-test) from analysing the qPCR data. Blue and red dots are genes downregulated and upregulated, respectively. Dark and light colors (blue or red) indicate *P* value < 0.05 and > 0.05, respectively.

Supplementary Figure 2. MRP4 is correlated with β-catenin in other cancers.

Correlation analysis of MRP4 and β-catenin mRNA levels in human colorectal cancer (A-C, GSE24551, GSE24549 and GSE75315, http://www.ncbi.nlm.nih.gov/geo), colon adenocarcinoma (D, TCGA-ID: COAD, http://cancergenome.nih.gov/), prostate cancer (E-F, GSE46691 and GSE21034, http://www.ncbi.nlm.nih.gov/geo), bladder cancer (G, GSE57933, http://www.ncbi.nlm.nih.gov/geo) and breast cancer (H, GSE12093, http://www.ncbi.nlm.nih.gov/geo). n is indicated for each dataset. Values of *r* and *P* are shown for each analysis by Pearson correlation test.

Supplementary Figure 1

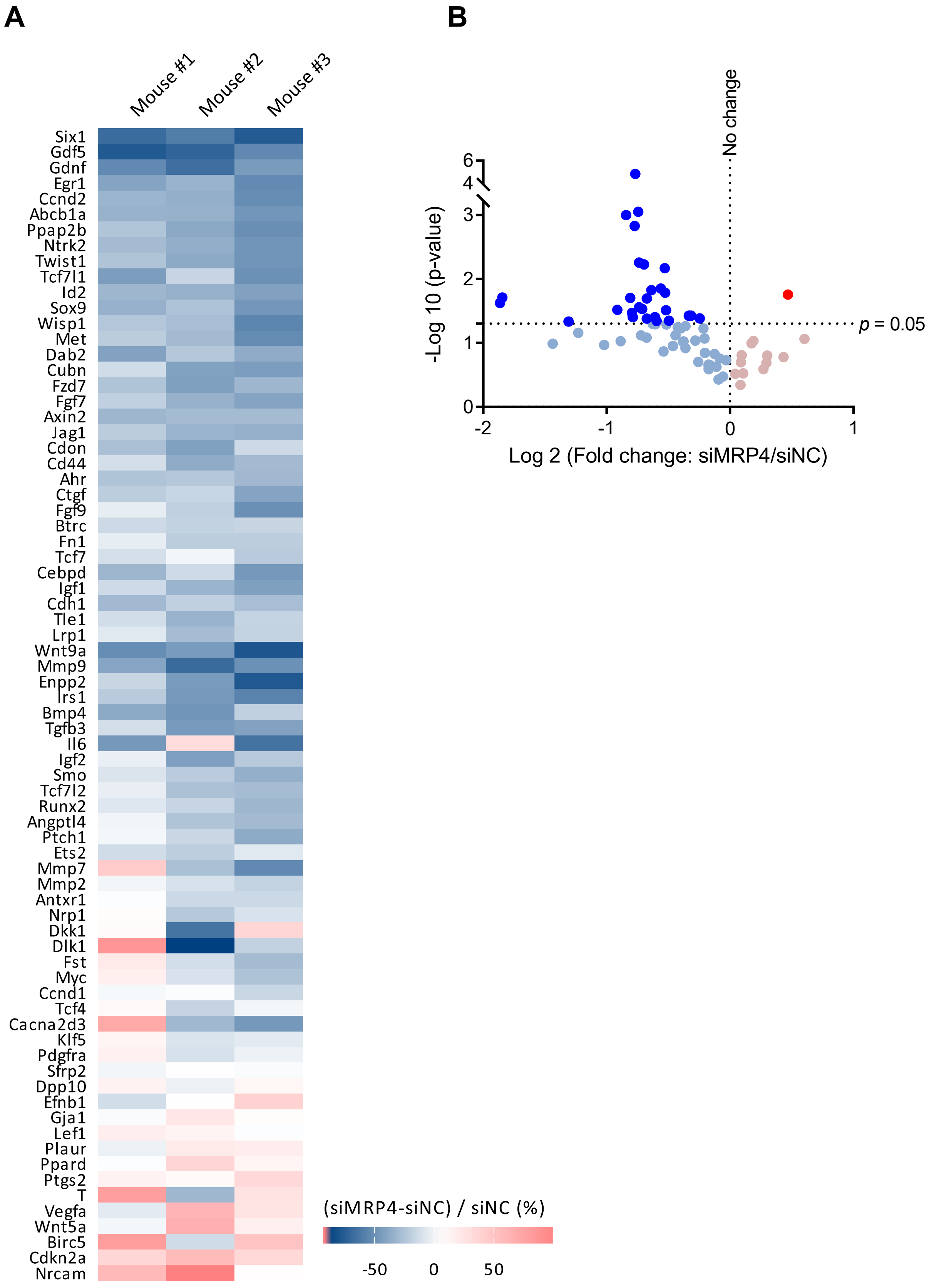

Supplementary Figure 2

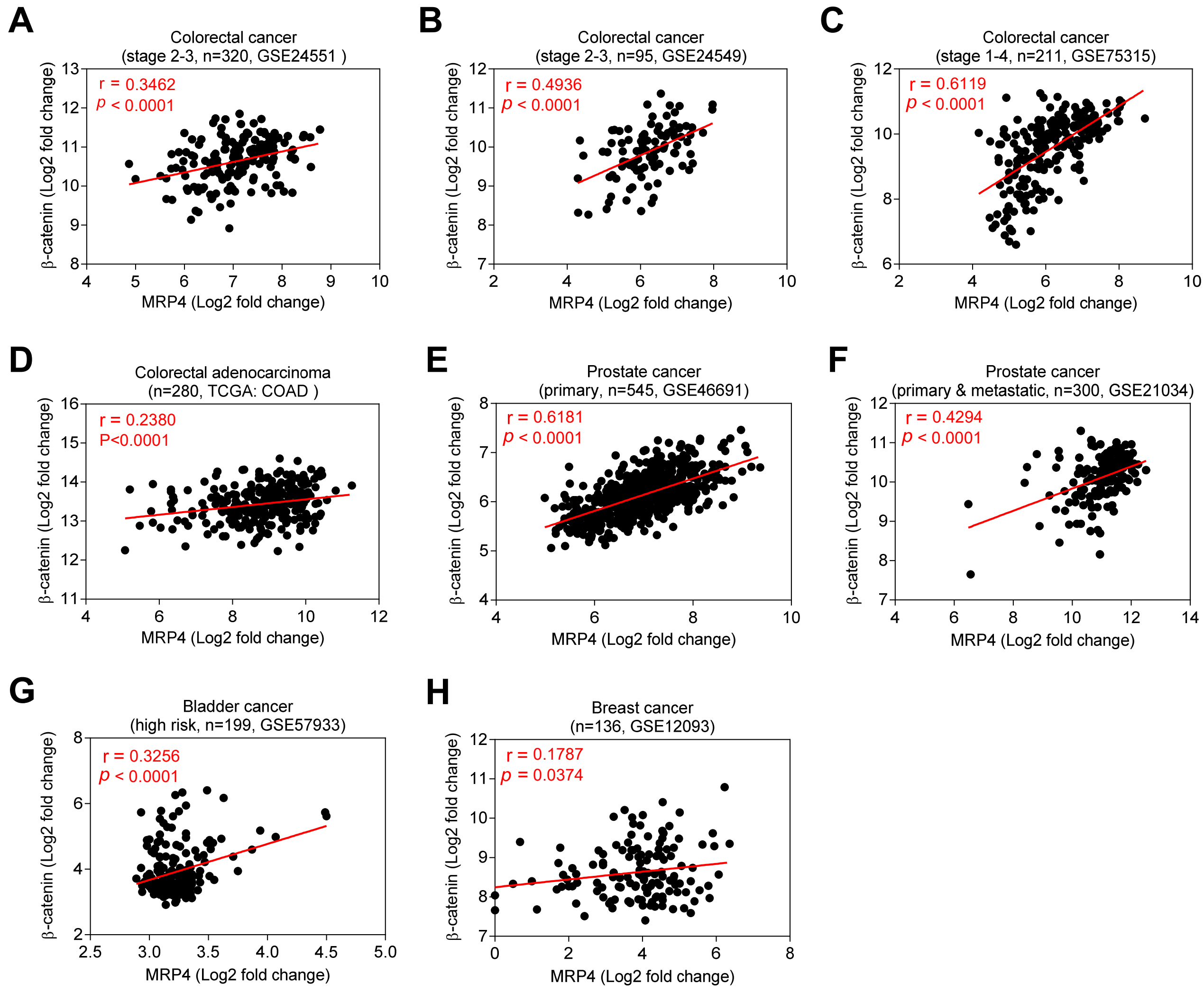

Supplement: Supplementary file 1 — Supplementary figures. [file thnov09p5049s1.pdf]
